# Pathway analysis reveals functional convergence of gene expression profiles in breast cancer

**DOI:** 10.1186/1755-8794-1-28

**Published:** 2008-06-27

**Authors:** Ronglai Shen, Arul M Chinnaiyan, Debashis Ghosh

**Affiliations:** 1 Department of Epidemiology and Biostatistics, Memorial Sloan-Kettering Cancer Center, New York, NY, USA; 2 Department of Pathology and Urology, University of Michigan, Ann Arbor, MI, USA; 3 Departments of Statistics and Public Health Sciences, Penn State University, University Park, PA, USA

## Abstract

**Background:**

A recent study has shown high concordance of several breast-cancer gene signatures in predicting disease recurrence despite minimal overlap of the gene lists. It raises the question if there are common themes underlying such prediction concordance that are not apparent on the individual gene-level. We therefore studied the similarity of these gene-signatures on the basis of their functional annotations.

**Results:**

We found the signatures did not identify the same set of genes but converged on the activation of a similar set of oncogenic and clinically-relevant pathways. A clear and consistent pattern across the four breast cancer signatures is the activation of the estrogen-signaling pathway. Other common features include BRCA1-regulated pathway, reck pathways, and insulin signaling associated with the ER-positive disease signatures, all providing possible explanations for the prediction concordance.

**Conclusion:**

This work explains why independent breast cancer signatures that appear to perform equally well at predicting patient prognosis show minimal overlap in gene membership.

## Background

Many studies have demonstrated the ability of using gene-expression "signatures" derived from DNA microarray data to define cancer subtypes, predict disease recurrence, and guide treatment decisions. In breast cancer, van't Veer *et al*. [1] derived a 70-gene profile to predict a patient's risk of developing distant metastases. Perou *et al*. [2] and Sorlie *et al*. [3] developed an intrinsic-subtype signature that classifies breast tumors into molecular subtypes showing distinct differences in prognosis. From a cancer biology perspective, Chang *et al*. [4] studied the links between the wound healing process and cancer progression. Based on the expression pattern of a wound-response signature of 512 genes, they classify a tumor to have either activated or quiescent response and found this to be a significant prognostic predictor of tumor metastasis. These are promising results and a few of these signatures have begun to be assessed in clinical settings. Two questions have often been asked: (1) are these signatures identifying the same set of genes and (2) will they generate similar prediction performance when tested in new data sets?

The answer to the first question has been discouraging. Any pair of these signatures share only a few common genes. Possible reasons have been suggested including the differences in patient cohort characteristics (such as the distribution of age or stage of the disease), lack of comparability and reproducibility of the data generated using different microarray platforms, and varying statistical procedures used to generate the gene list. Nevertheless, Ein-Dor *et al*. [5] showed that the inconsistency still exists when eliminating all three differences. In particular, the authors repeated the same analysis in a single data set and identified many lists of genes equally predictive of the outcome. Any two of these gene lists share only a small number of genes. In another study by Son *et al*. [6], the authors reported that any randomly selected subgroup of around 100 differentially expressed genes generates similar hierarchical clustering results in the same data set.

Ein-Dor *et al*. [7] further suggested perhaps the main source of the problem lies in the small sample size and large number of genes the signatures were derived from. For several published breast cancer data sets, the authors estimated that several thousands of samples would be needed to achieve a typical gene overlap of 50%. On the other hand, the problem is compounded by analyzing and interpreting genes in isolation. A common approach to gene selection involves selecting a handful of top-ranking genes that best differentiate sample classes (such as tumor vs. normal tissue) or are most predictive of clinical outcome. The univariate selection procedure ignores correlation between genes. The biological and statistical validity of such assumption seems tenuous. As a result, gene-set based approaches have emerged in recent years to identify sets of biologically related genes that are deregulated as a group. Examples of gene-set analysis include the Gene Set Enrichment Analysis (GSEA) [8], Significance Analysis of Function and Expression (SAFE) [9], and the globaltest package [10] These methods focus on groups of genes that share common biological functions such as cell cycle regulation; metabolic or signaling pathways defined by Gene Ontology (GO); online databases such as BioCarta, KEGG and signaling data base; or a literature-defined gene set subject to experimental perturbations such as a drug treatment or an oncogene-activation. In addition, Rhodes *et al*. [11] introduced a Molecular Concepts Map (MCM) providing an expanded analytic framework to explore the network of relationships among biologically related gene sets.

The motivation of this study came from a recent paper by Fan *et al*. [12], which addresses the second question described above. The authors demonstrated a high degree of prediction concordance of five breast cancer gene-signatures despite minimal gene-wise overlap. In an independent data set of 295 tumors, the authors showed that the intrinsic subtypes [3] of basal-like, HER2+/ER-, and luminal B were consistently classified as poor 70-gene profile [13] prognosis, activated wound response [4] and high recurrence score [14]. It raises the question that perhaps the gene-overlap is not the most relevant measure of robustness and reproducibility of the gene-signatures. There may be common themes shared across these signatures that are not apparent on the individual gene level. As an example, the cell cycle gene Cyclin E1 (CCNE1) was included in the 70-gene profile while Cyclin E2 (CCNE2) in the intrinsic subtype signature. The two signatures apparently share commonality in the activation of the Cyclin family genes. For another example, ERBB2 and EGFR are both receptor tyrosine kinase involved in estrogen pathway. Inclusion of one or the other in two different signatures apparently converges at the pathway level both indicating the activation of the estrogen-signaling pathway.

In this study, we assess the potential functional convergence of these gene-signatures on the basis of activated oncogenic pathways. This involves first annotating each gene-signature to identify significantly enriched functional modules (e.g., cell growth, response to estrogen, myb-regulated pathways, etc.). Definition of the modules can be based on Gene Ontology (GO) terms, online pathway databases such as BioCarta and KEGG, or literature-defined concepts. In the next step, the overlapping functional modules are obtained by intersecting the annotated sets. We investigated six breast cancer signatures (four of which were compared in Fan *et al*. [12]) that share high prediction concordance. We found eighteen common modules including estrogen-signaling, responses to tamoxifen treatment, and BRCA1 expression. The degree of the functional overlap across the six BR-signatures is highly significant (P = 0.0002) under a bootstrapped null distribution.

## Results and Discussion

### Prediction concordance across five breast-cancer gene-signatures

In a similar fashion as in [12], we cross-tabulated the prediction results of the gene-signatures listed in Table 1 in the 295 breast cancer patients in the van de Vijver study [15]. In Table 2, all the signatures consistently classified the basal-like and HER2/ER- subtype tumors as having high risk of recurrence outcome. The 70-gene profile and wound-response signatures both classify luminal B subtype to be a low risk group, while the meta-signature classifies the luminal A and the normal-like subtypes as low risk groups. Overall, the signatures showed a certain degree of prediction concordance. The kappa coefficient measuring the classification agreement across the signatures is estimated to be 0.67.

**Table 1:** Breast cancer gene-signatures.

**Gene-signature**	**Number of genes**	**number of samples**	**Experiment summary**
1. 70-gene profile [1]	70	78	Inkjet oligonucleotide array on 25,000 genes
2. Wound-response [4]	512	50	cDNA microarrays profiled over 36,000 genes
3. Intrinsic subtype [2,3]	427	78	cDNA microarrays on a core set of 8,102 genes
4. meta-90 [11]	90	305	Integrative analysis of 4 microarray studies on a set of 2,555 genes
ER+ signature			
5. Recurrence score [14]	21	2892	RT-PCR on 250 genes selected from the literature
6. Wang ER+ profile [18]	60	80	Affymetrix GeneChips on 22,000 transcripts

**Table 2:** Classification concordance of the breast cancer gene signatures (Kappa coefficient = 0.67)

**Intrinsic Subtype**	**No. of Patients**	**70-Gene Profile**		**Wound Response**		**Meta90**	
	Patients	Classification	No. of Patients	Classification	No. of Patients	Classification	No. of Patients

Basal-like	36	Good	0	Quiescent	0	Low	0
		Poor	36	Activated	36	High	36
Luminal A	91	Good	69	Quiescent	34	Low	89
		Poor	22	Activated	57	High	2
Luminal B	41	Good	5	Quiescent	1	Low	16
		Poor	36	Activated	40	High	25
HER2+ and ER-	28	Good	3	Quiescent	0	Low	8
		Poor	25	Activated	28	High	20
Normal-like	23	Good	12	Quiescent	12	Low	22
		Poor	11	Activated	11	High	1

### Common "oncogenic" sets underpinning breast cancer outcome prediction

**Figure 1:**
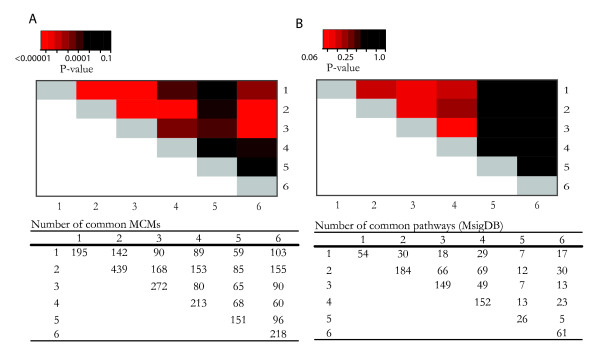
**Pair-wise functional overlap of the six breast cancer gene-signatures**. 1. 70-gene profile 2. Wound response 3. Intrinsic subtype 4. Meta90 5. Recurrence score 6. Wang ER+ profile. A. The number of overlapping literature-defined oncogenic concepts (MCM) and the corresponding P-value heatmap indicating the significance of the overlap under bootstrapped null distribution. B. The number of overlapping pathway sets (MsigDB) and the corresponding P-value heatmap.

For pairs of the six signatures, there is a fair amount of overlapping literature concepts (MCMs). Many of the overlaps are highly significant (Figure 1A). For example, there is a set of 142 enriched MCM modules shared between the 70-gene profile and the wound-response signature (*P *< 0.00001) while only two genes were identified by both. Furthermore, signatures 1–3 showed marginal significance in metabolic and signaling pathway overlaps (Figure 1B).

We found 18 common MCMs (*P *= 0.0002) and 5 common metabolic and signaling pathways (*P *= 0.04) across signatures 1–4. Table 3 and 4 list these common sets ordered by the overall significance of enrichment (summarized hypergeometric test P-value adjusted for multiple testing). Among the top are deregulated genes in androgen-sensitive prostate cancer cell lines in response to MSA (MCM 258), Myb-regulated transcriptional changes in the estrogen-dependent human breast cancer cell line MCF7 (MCM 458), several MCMs comprising responsive genes upon antiestrogen hormonal treatment (MCM 691, 379, 375, 673). Clearly a dominant common characteristic underpinning the four breast-cancer signatures is closely related to the estrogen-receptor status of the tumor which is a main prognostic factor in breast cancer. Another common prognostic set of interest is response to BRCA1 expression (MCM513), which many studies have shown a characteristic of sporadic basal-like cancers.

**Table 3:** Eighteen common literature-defined oncogenic concepts (MCM) across the four breast cancer gene signatures (significance of overlap, P = 0.0002)

	**70-gene profile**	**Wound Response**	**Intrinsic Subtype**	**Meta-90**		
**Common GeneSet**	No. of mapped genes (Enrichment p-value**)				**MCM size**	**Description**

MCM258	7 (3e-04)	25 (2e-04)	19 (0.21)	6 (0.09)	350	Downregulated genes in prostate cancer cells in response to MSA (full list)
MCM458	2 (0.16)	24 (1e-04)	34 (0.005)	6 (0.24)	322	Differentially expressed genes in MCF7 cells expressing Myb
MCM396	10 (0.13)	89 (2e-04)	117 (0.005)	20 (0.21)	2265	Upregulated genes in U937 cells expressing the PLZF/RAR fusion protein
MCM691	1 (0.1)	6 (0.06)	19 (6e-04)	3 (0.16)	101	Up-regulated genes in untreated or permanently tamoxifen-treated MaCa 3366/TAM compared with MaCa 3366
MCM513	2 (0.22)	24 (6e-04)	29 (0.13)	12 (0.04)	375	Differentially expressed genes in EcR-293 cells in response to BRCA1 expression
MCM277	1 (0.01)	3 (0.02)	5 (0.05)	1 (0.16)	22	Upregulated genes in NCCIT cells in response to Wnt-3A
MCM30	1 (0.01)	4 (0.004)	3 (0.25)	1 (0.15)	24	Upregulated genes in colorectal cancer cells
MCM673	2 (0.01)	7 (0.008)	7 (0.28)	3 (0.15)	79	Androgen
MCM6209872	2 (0.003)	2 (0.13)	5 (0.09)	1 (0.16)	34	Skin
MCM349	1 (0.01)	2 (0.05)	2 (0.25)	2 (0.04)	23	Downregulated genes in hSNF5/INI1-deficient malignant rhabdoid tumor cell line upon hSNF5/INI1 expression
MCM12	2 (0.008)	2 (0.25)	7 (0.09)	2 (0.14)	56	Aniogenic and Non-angiogenic tumours Signature
MCM363	5 (0.13)	37 (0.007)	46 (0.28)	14 (0.15)	808	Upregulated genes in monocytes in response to IL-10 stimulation for 1 and 4 hours
MCM574	4 (0.04)	22 (0.03)	23 (0.27)	6 (0.13)	497	Upregulated genes in advanced papillary serous tumor specimens
MCM683	1 (0.1)	6 (0.06)	11 (0.06)	2 (0.17)	111	Downregulated genes wrt 3,5-diaryl-1,2,4-oxadiazole (MX-126374)
MCM379	1 (0.13)	7 (0.07)	12 (0.12)	2 (0.29)	129	Unique genes regulated by tamoxifen, but not estradiol in osteosarcoma cells
MCM1067	1 (0.05)	3 (0.15)	5 (0.27)	1 (0.21)	64	Upregulated genes in immmortilized epithelial cells in respense to Ad5-GFP infection
MCM375	1 (0.13)	6 (0.12)	10 (0.14)	2 (0.27)	127	Unique genes regulated by estradiol, but not raloxifene in osteosarcoma cells
MCM402	1 (0.11)	4 (0.25)	8 (0.28)	3 (0.14)	116	Downregulated genes in HepG2 T1 treated cells resulting from MIZ depletion

**Hypergeometic test enrichment P-value adjusted for multiple testing.

**Table 4:** Five common pathway sets (MsigDB) across the four breast cancer gene signatures (significance of overlap, *P *= 0.04).

	**70 Gene**	**Wound Response**	**Intrinsic Subtype**	**Meta90**	
**Common GeneSet**	No. of mapped genes (Enrichment p-value**)				**Description**

breast cancer estrogen signaling	1 (0.07)	11 (0.001)	11 (0.22)	4 (0.11)	GEArray
EMT DOWN	1 (0.02)	2 (0.19)	4 (0.29)	1 (0.21)	Jechlinger et al 2003
CR DNA MET AND MOD	1 (0.01)	1 (0.24)	3 (0.24)	1 (0.12)	PNAS 2007
SA REG CASCADE OF CYCLIN EXPR	1 (0.006)	1 (0.12)	2 (0.23)	1 (0.07)	SigmaAldrich
reckPathway	1 (0.005)	1 (0.09)	1 (0.28)	1 (0.09)	BioCarta

** Hypergeometic test enrichment P-value adjusted for multiple testing.

Table 4 listed the five common metabolic and signaling pathways using the functional subset of the MsigDB annotation data. All of the signatures apparently enlisted genes customized on a commercial array platform that represent the breast cancer estrogen signaling pathway [see Additional File 1].

For the gene signatures listed in Table 1, it should be pointed out that they were constructed using different types of endpoints, along with differing supervised learning algorithms. In attempting to combine results across the signatures, we make the assumption that there exists an underlying tumorigenetic mechanism that manifests itself in terms of the endpoints used by the different authors. One such mechanism might be tumor metastasis.

### ER-positive relapse signatures

Both ER+ relapse-signatures showed evidence of E2F activation, response to Interleukin-6 (IL6), and activation of insulin-signaling pathways, some of which have been reported in the literature to be specific to ER+ disease [see Additional Files 2 and 3]. For example, studies have shown in estrogen-sensitive breast cancer cell lines, the widely used antiestrogen tamoxifen treatment inhibits insulin-signaling. The degree of such inhibition can reflect the effectiveness of the tamoxifen treatment and thus correlate with a patient's risk of recurrence [16,17].

## Conclusion

Cancer gene-expression signatures derived from microarray experiments are beginning to be tested in clinical trials, while the exact biology that enables these gene-signatures to accurately predict tumor metastasis and patient survival is unclear. Microarray experiments are often limited in power by the small number of samples used to derive a panel of prognostic genes relative to the large number of features on the array. In addition, sets of biologically-related genes are often co-regulated while many feature selection procedures are univariate in nature. As a result, gene-signatures developed by different studies typically share very few common components. A recent study showed high prediction concordance of several breast cancer gene-signatures despite minimal overlap in gene identity. It gave main motivation to investigate common oncogenic themes that may not be apparent at the individual gene level. This study explored this hypothesis by evaluating the functional overlap of the signatures on the basis of annotated gene sets. When the gene signatures are mapped to the deregulated pathway space, two things become clear. First, there is a significant degree of functional overlap in oncogenic and prognostic pathways. Second, many of these common pathways provide plausible explanations of tumor biology through which these signatures predict patient outcome. There are several conclusions to be gleaned from this study. First, this work explains why independent signatures that appear to perform equally well at predicting patient prognosis show minimal overlap in gene membership. This is because such genes are different members of pathways and processes that are relevant to prognosis. Thus, the lack of gene overlap found between the various signatures listed in Table 1 should not be considered problematic. The implication of our study is that most of these signatures will do well in clinical trials given that they seem to be picking up the same pathway signals. We can thus be assured that the gene lists found by different investigators are consistent, even if they do not contain the same genes.

Second, the results have suggested that the interpretability and delineation of how diverse cancer gene expression signatures work are more likely attainable at the pathway level rather than the individual gene level. On the other hand, as many studies have already suggested so, feature selection methods need to be based on biologically related gene sets that are deregulated as a group [8-11]. However, it is not a straightforward task to construct a prognostic signature based on pathways that are composed of overlapping sets of genes. New statistical methods need to be established in this area. This is beyond the scope of the study and is currently under investigation.

## Methods

Table 1 lists the six BR-signatures that are compared in this study. Fan *et al*. [10] showed high prediction concordance of signature 1–3 and signature 5. In addition, a 90-gene meta-signature [13] is included. This signature was derived in a meta-analysis framework by integrating four microarray data sets, which included the van't Veer data set and the Sorlie data set. Another signature included here is the subset of 60-gene profile from Wang *et al*. [18] that was derived in tumors with estrogen receptor (ER) positive status. The recurrence-score signature [14] is also an ER+ disease signature that has been shown in a clinical trial to be able to identify patients with very low risk of recurrence on hormone therapy using tamoxifen alone, and do not require adjuvant chemotherapy.

### Annotation

The set of signature genes were annotated using two different annotation sources:

#### Literature-defined module

A collection of 661 literature-defined modules from the Molecular Concept (MCM) database MCM that focuses on human cancer studies. These include gene sets from peer-reviewed publications using microarrays to study gene expression changes subject to experimental perturbation such as drug treatment or candidate gene activation.

#### Pathway module

The functional subsets from the molecular signature database or MSigDB GSEA, including modules representing metabolic and signaling pathways imported from online pathway databases such as BioCarta [19], signalling pathway database [20] and the Kyoto Encyclopedia of Genes and Genomes (KEGG) [21].

Enrichment analysis was performed using hypergeometric tests. In particular, the procedure tests the significance of the proportion of module genes (e.g., estrogen pathway) in the signature being greater than the "population"-proportion of the module genes in the experimental set from which the signature was selected. Multiple testing was adjusted by using the Benjamini-Hochberg procedure [22].

### Notation and methods

For a set of *K *gene-signatures, let *n_*i *_*be the number of genes in signature *i *and *N_*i *_*be the total number of genes in the experimental set from which the signature genes were selected. Furthermore, let *J *= 661 or 552 denote the number of literature-defined concepts and the number of metabolic and signaling pathways in the two annotation database MCM and MsigDB respectively. For a gene signature, we first perform a module enrichment analysis using a hypergeometric test. As mentioned earlier, the basic idea is to test whether the proportion of the module genes in the signature of size *n_*i *_*is significantly larger than the "population"-fraction of the module genes in the experimental set of size *N_*i*_*. The *j*th module is enriched in the *i*th signature if the hypergeometric test p-value is less than 0.3. Across the *K *signatures under comparison, this threshold correspond to a p-value of less than 0.05 under a conventional meta-analysis of combining the hypergeometric p-values $-2\sum_{i=1}^{K}\log P_{i}$ across the four signatures based on a chi-square distribution with 2*K *degrees of freedom. Let *X_*ij *_*be the indicator variable where *X*_*ij *_= 1 if the *j*th module is enriched in the *i*th (*i *= 1, ..., *K*) signature and *X*_*ij *_= 0 otherwise. As a result,

$$m_{i}=\sum_{j=1}^{J}X_{ij}$$

is the total number of enriched modules in signature *i*. Then for the set of *K *signatures, the amount of functional overlap is $Y=\sum_{j=1}^{J}\prod_{i=1}^{K}X_{ij}$.

The significance of overlap is defined as *P *(*Y *> *y*^*obs*^) under a bootstrapped null distribution. The bootstrap procedure is described elsewhere [see Additional File 4].

We used *B *= 100,000 in the procedure. The bootstrapped null distribution of *Y *preserves 1) potential correlation of the signature size *n_*i *_*and the number of enriched modules *m_*i*_*, and 2) the module-module dependence due to the one-to-many mapping of a gene to the annotation data.

## Competing interests

The authors declare that they have no competing interests.

## Authors' contributions

DG and RS conceived the method and prepared the manuscript. RS performed the analyses. AC contributed to the discussion. All authors have read and approved the final manuscript.

## Pre-publication history

The pre-publication history for this paper can be accessed here:



## Supplementary Material

Additional file 1 List of modules genes involved in A. estrogen signaling and B. response to MSA in androgen-dependent prostate cell lines. Click here for file

Additional file 2 List of the fifty-two common MCM sets shared between the two ER+ gene-signatures. Click here for file

Additional file 3 List of the five common metabolic and signaling pathway sets (MsigDB) shared between the two ER+ gene-signatures. Click here for file

Additional file 4 Description of algorithm used to test for significance of overlap of datasets. Click here for file
