# Supplementary material for: Pathway analysis reveals functional convergence of gene expression profiles in breast cancer
Source: BMC Med Genomics. 2008 Jun 27;1:28. doi: 10.1186/1755-8794-1-28 (PMC2447843; doi:10.1186/1755-8794-1-28)
Supplement: Additional file 1 — List of modules genes involved in A. estrogen signaling and B. response to MSA in androgen-dependent prostate cell lines. [file 1755-8794-1-28-S1.doc]

**List of module genes.**

| **A. Breast cancer estrogen signaling** | | |  |  |  |  |  |
| --- | --- | --- | --- | --- | --- | --- | --- |
| **Signature** | **Module genes** | |  |  |  |  |  |
| 1. 70-gene profile | CCNE1 |  |  |  |  |  |  |
| 2. Wound-response | CDKN1A | GSN | IL6ST | KIT | TNFAIP2 | NME1 |  |
|  | F3 | HMGB1 | ID2 | ITGA6 | MT3 |  |  |
| 3. Intrinsic subtype | ERBB2 | PPP1R15A | KIT | CDH1 | CCNE1 | GABRP |  |
|  | IGFBP2 | CCND1 | MUC1 | GATA3 | ESR1 |  |  |
| 4. Meta90 | GATA3 | BCL2 | VEGF | CCNE1 |  |  |  |
| **B. MCM 258: Downregulated gene in prostate cancer cells in response to MSA** | | | | | | |  |
| **Signature** | **Module genes** | |  |  |  |  |  |
| 1. 70-gene profile | GPR126 | MCM6 | DTL | RFC4 | CENPA | NUSAP1 | NDC80 |
| 2. Wound-response | BRIP1 | NUP35 | FADS2 | ACSL3 | MCM7 | MCM3 | WDHD1 |
|  | SCD | ACIN1 | INSIG1 | PPAN | RFC3 | CHEK1 | CDK2 |
|  | RBMX | HMGN2 | H2AFV | CENPN | BRCA2 | TMEM48 |  |
| 3. Intrinsic subtype | KCTD15 | SLC44A2 | PNKD | DSC2 | MCM3 | NQO1 | MCCC2 |
|  | INPP4B | CIT | RFC3 | ACAA2 | PRKACB | CLIP4 | CCND1 |
|  | WNK1 | APOD | CAMK2N1 | STEAP2 | ZNF532 |  |  |
| 4. Meta90 | TIMP1 | SLC25A1 | CSE1L | CDKN3 | HMMR | GEMIN4 |  |
