# Supplementary material for: Pathway analysis reveals functional convergence of gene expression profiles in breast cancer
Source: BMC Med Genomics. 2008 Jun 27;1:28. doi: 10.1186/1755-8794-1-28 (PMC2447843; doi:10.1186/1755-8794-1-28)
Supplement: Additional file 2 — List of the fifty-two common MCM sets shared between the two ER+ gene-signatures. [file 1755-8794-1-28-S2.doc]

| List of the fifty-two common MCM sets shared between the two ER+ gene-signatures (significance of overlap, P=0.3). | | | | |
| --- | --- | --- | --- | --- |
|  | **Recurrence-score** | **Wang ER+ profile** |  |  |
| **Common GeneSet** | **No. of mapped genes (Enrichment P-value**)** | | **size** | **Description** |
| MCM505 | 1 (0) | 1 (0.005) | 19 | Upregulated genes in MDMs in response to gp120 stimulation for 6 hours |
| MCM542 | 1 (0) | 2 (2e-04) | 13 | Upregulated genes in melanocytes in response to SCF that are Mitf dependent and resistant to cycloheximide |
| MCM677 | 1 (0.05) | 3 (0) | 15 | Downregulated genes in Gastric Cancer Cell Line Treated with Trichostatin A |
| MCM260 | 5 (0.04) | 7 (4e-04) | 501 | Downregulated genes (time dependent) in prostate cancer cells in response to Resveratrol |
| MCM7 | 3 (0.06) | 4 (7e-04) | 189 | Differentially expressed genes in HeLa cells during the cell cylce- G2/M Phase |
| MCM538 | 2 (0.08) | 4 (5e-04) | 174 | Upregulated genes in U2OS cells upon E2F2 expression |
| MCM373 | 1 (0.03) | 2 (0.002) | 42 | Upregulated genes in kidney epithelial cells in response to PLAG1 expression |
| MCM6209902 | 3 (0.16) | 4 (7e-04) | 144 | knockdownRb1 |
| MCM487 | 3 (0.1) | 3 (0.001) | 93 | Upregulated genes in myeloma cells in response to IL-6 treatment |
| MCM297 | 2 (0.02) | 1 (0.007) | 26 | Downregulated genes in SWB40 Astrocytoma cells in response to methionine |
| MCM681 | 1 (0.03) | 1 (0.007) | 30 | Downregulated genes in Gastric Cancer Cell Line Treated with Trichostatin A / AdoHcy |
| MCM584 | 9 (0.1) | 16 (0.002) | 2588 | Upregulated genes in CD4 cells compared to other blood cells |
| MCM496 | 3 (0.08) | 3 (0.003) | 141 | Genes in myeloma cells with similar expression in response to IL-6 treatment and to stromal stimulation |
| MCM573 | 1 (0.05) | 1 (0.007) | 26 | Upregulated genes in MCLs compared with normal B-cell populations |
| MCM293 | 1 (0.08) | 1 (0.004) | 18 | Downregulated genes in DAOY Medulloblastoma in response to methionine |
| MCM295 | 1 (0.05) | 1 (0.007) | 30 | Downregulated genes in SWB61 Oligodendroglioma in response to methionine |
| MCM471 | 2 (0.03) | 2 (0.02) | 166 | Upregulated genes in HUVECs in response to Egr-1 expression |
| MCM536 | 3 (0.05) | 4 (0.008) | 484 | Upregulated genes in U2OS cells upon E2F1 expression |
| MCM395 | 6 (0.1) | 12 (0.004) | 2270 | Downregulated genes in U937 cells expressing the PLZF/RAR fusion protein |
| MCM490 | 4 (0.03) | 1 (0.02) | 66 | Downregulated genes in myeloma cells in response to a constitutiveexpression of N-ras vs.IL-6 treatment |
| MCM567 | 4 (0.02) | 1 (0.03) | 89 | Differentially expressed genes in MM cells in response to SAHA |
| MCM683 | 3 (0.09) | 2 (0.008) | 111 | Downregulated genes wrt 3,5-diaryl-1,2,4-oxadiazole (MX-126374) |
| MCM583 | 4 (0.09) | 6 (0.01) | 797 | Upregulated genes in basophils compared to other blood cells |
| MCM236 | 1 (0.27) | 3 (0.004) | 195 | Upregulated genes related to aging in kidneys excluding those with higher expression in blood |
| MCM384 | 1 (0.05) | 1 (0.02) | 67 | Downregulated genes in TK6 cells upon MNNG treatment |
| MCM92 | 5 (0.06) | 5 (0.02) | 718 | Top-ranked UV-repressed genes in acute radiation toxicity |
| MCM206 | 1 (0.13) | 1 (0.01) | 37 | Upregulated genes in good kidney transplants compared to donor |
| MCM385 | 2 (0.05) | 1 (0.03) | 94 | Upregulated genes in TK6 cells with a mutated hMSH6 gene |
| MCM386 | 2 (0.05) | 1 (0.03) | 94 | Downregulated genes in TK6 cells with a mutated hMSH6 gene |
| MCM95 | 4 (0.14) | 4 (0.01) | 460 | Top-ranked IR-induced genes in acute radiation toxicity |
| MCM350 | 1 (0.13) | 1 (0.01) | 50 | Upregulated genes in hSNF5/INI1-deficient malignant rhabdoid tumor cell line upon hSNF5/INI1 expression |
| MCM240 | 2 (0.16) | 2 (0.01) | 178 | Upregulated genes in HEK cell lines in response to SV40 small tumor antigen (ST) |
| MCM66 | 2 (0.18) | 2 (0.01) | 150 | Upregulated in Wilm‘s Tumor |
| MCM242 | 2 (0.06) | 1 (0.04) | 126 | Upregulated genes in HEK cell lines in response to COOH terminal deletion mutant SV40 small tumor antigen (ST) |
| MCM78 | 2 (0.28) | 4 (0.009) | 427 | Upregulated genes by IFN-alpha in hepatitis C- Full List |
| MCM391 | 3 (0.1) | 5 (0.03) | 967 | Downregulated genes in U937 cells expressing the AML1/ETO fusion protein |
| MCM73 | 1 (0.2) | 1 (0.01) | 48 | Downregulated genes in systemic lupus erythematosus |
| MCM707 | 4 (0.03) | 1 (0.1) | 199 | Downregulated genes in neuroblastoma cells in response to BL1521 treatment |
| MCM713 | 2 (0.06) | 1 (0.05) | 121 | Translationally reepressed genes in Jurkat T cells in response to rapamycin treatment |
| MCM494 | 1 (0.23) | 3 (0.01) | 277 | Upregulated genes in response to stromal stimilation, IL-6 treatment, and constitutive expression of N-ras |
| MCM143 | 1 (0.23) | 1 (0.01) | 50 | Downregulated genes by ICI, Ral, or TOT, but not estradiol |
| MCM673 | 1 (0.13) | 1 (0.03) | 79 | Androgen |
| MCM435 | 1 (0.13) | 1 (0.03) | 79 | Upregulated genes in PMNs upon migration to skin lesions |
| MCM497 | 1 (0.16) | 1 (0.03) | 81 | Upregulated genes in OEA‘s as a result of deregulation of Beta-catenin |
| MCM590 | 4 (0.06) | 2 (0.08) | 365 | Upregulated genes in melanocytes in response to stem cell factor (SCF) stimulation |
| MCM7621582 | 1 (0.1) | 2 (0.07) | 370 | Blood |
| MCM575 | 2 (0.12) | 1 (0.08) | 168 | Downregulated genes in the Dox6 MDR multiple myeloma cell line |
| MCM22 | 1 (0.23) | 1 (0.05) | 117 | Downregulated genes in Pancreatic cancer cells with the methylase inhibitor 5-aza-2‘-deoxycytidine (5-aza-CdR) |
| MCM161 | 1 (0.23) | 1 (0.05) | 140 | Upregulated genes in prostate cancer after androgen ablation therapy |
| MCM94 | 4 (0.11) | 3 (0.1) | 661 | Top-ranked IR-repressed genes in acute radiation toxicity |
| MCM565 | 4 (0.16) | 1 (0.16) | 259 | Downregulated genes in fibroblasts expressing the EWS/FLI fusion protein |
| MCM587 | 4 (0.11) | 2 (0.26) | 668 | Upregulated genes in eosinophils compared to other blood cells |
