# Supplementary material for: Pathway analysis reveals functional convergence of gene expression profiles in breast cancer
Source: BMC Med Genomics. 2008 Jun 27;1:28. doi: 10.1186/1755-8794-1-28 (PMC2447843; doi:10.1186/1755-8794-1-28)
Supplement: Additional file 3 — List of the five common metabolic and signaling pathway sets (MsigDB) shared between the two ER+ gene-signatures. [file 1755-8794-1-28-S3.doc]

| List of the five common metabolic and signaling pathway sets (MsigDB) in ER+ gene-signatures (significance of overlap, P=0.5). | | | |
| --- | --- | --- | --- |
|  | **Recurrence-score** | **Wang ER+ profile** |  |
| **Common GeneSet** | **No. of mapped genes (Enrichment q-value)** | | **Description** |
| INSULIN 2F UP | 1 (0.17) | 2 (0.01) | Rome et al 2003 |
| RAP DOWN | 1 (0.21) | 1 (0.09) | Peng et al 2002 |
| LEU DOWN | 1 (0.2) | 3 (0.002) | Peng et al 2002 |
| PGC | 1 (0.24) | 1 (0.23) | Manually Curated |
| insulin signaling | 1 (0.3) | 1 (0.03) | BioCarta |
