# Supplementary material for: Pathway analysis reveals functional convergence of gene expression profiles in breast cancer
Source: BMC Med Genomics. 2008 Jun 27;1:28. doi: 10.1186/1755-8794-1-28 (PMC2447843; doi:10.1186/1755-8794-1-28)
Supplement: Additional file 4 — Description of algorithm used to test for significance of overlap of datasets. [file 1755-8794-1-28-S4.rtf]

Algorithm 1 Test significance of overlap	
1: for b=1,...,B do	
2: Generate a set of pseudo-signatures  of size   by randomly sample (with replacement) from the experimental sets  of size.	
3: Hypergeometric tests for significantly enriched functional modules. 	
4: Compute the degree of overlap , where  is the indicator variable of whether the jth module is enriched in pseudo-signature .	
5: end for	
6: Generate the empirical null distribution : probability mass 1/B on .	
7: return 	
